# A New Anæsthetic

**Published:** 1861-05

**Authors:** 


A NEW ANÆSTHETIC.—Dr. John Wilmhurst, of the ship Marathon, Cunard line, in a letter to the *Lancet*, states that he believes he has discovered a new anæsthetic agent and valuable anodyne in the rectified oil of turpentine. While on board the emigrant ship Indiana, he was applied to for severe supra-orbital neuralgia, and his stock of chloroform being exhausted, he sprinkled a few drops of the turpentine on a handkerchief, and applied it to the nostrils of the sufferer. After a few inhalations it produced a gentle sleep, from which the patient awoke, free from pain, and without headache or any unpleasant symptoms. He has also employed it in two cases of slight, but painful operations, as well as in cramps, renal calculus, and convulsions. "Its effect seems to be to allay nervous irritation, spasm, and pain, without deranging the action of the heart, and to produce a calm anæsthetic sleep."

